# Supplementary material for: The Role of Oxidative Stress in the Risk of Cardiovascular Disease and Identification of Risk Factors Using AIP and Castelli Atherogenicity Indicators in Patients with PCOS
Source: Biomedicines. 2022 Jul 14;10(7):1700. doi: 10.3390/biomedicines10071700 (PMC9312468; doi:10.3390/biomedicines10071700)
Supplement: Supplementary file 1 [file biomedicines-10-01700-s001.zip › biomedicines-1772868-supplementary.pdf]

Supplement 1

Comparison of anthropometric parameters in PCOS patients with and without IR.

	PCOS without IR *(n=21)*		PCOS with IR *(n=8)*		
Parameter	mean	SD	mean	SD	p
Age	26.33	7.35	29.25	6.25	0.172
Hight (m)	1.67	0.06	1.66	0.07	0.770
Weight (kg)	69.37	16.67	87.03	20.63	0.028
HIP (cm)	101.24	8.04	114.25	13.75	0.034
WC (cm)	80.43	16.27	100.63	16.00	0.006
Systolic pressure (mmHg)	115.48	13.12	127.50	14.99	0.083
Diastolic pressure (mmHg)	76.19	9.86	83.38	11.64	0.143
BMI (kg/m^2)	24.69	5.39	31.73	8.15	0.018
WHR	0.79	0.14	0.88	0.09	0.009

PCOS-polycystic ovary syndrome; IR-insulin resistance; BMI-body mass index; HIP-hip circumference; WC-waist circumference; WHR-waist-hip ratio; SD-standard deviation

Supplement 2.

Comparison of anthropometric parameters in patients with PCOS and hyperandrogenism and with PCOS without hyperandrogenism

	PCOS without HA *(n=14)*		PCOS with HA *(n=15)*		
Parameter	mean	SD	mean	SD	p
Age (year)	26.43	5.63	27.80	8.36	0.647
Hight (m)	1.65	0.05	1.55	1.71	0.074
Weight (kg)	68.81	16.11	79.31	20.99	0.176
HIP (cm)	102.29	10.97	107.20	11.52	0.295
WC (cm)	81.79	16.84	89.93	19.42	0.230
Systolic pressure (mmHg)	113.64	12.63	123.60	14.79	0.132
Diastolic pressure (mmHg)	75.36	10.46	80.80	10.54	0.230
BMI (kg/m^2)	25.28	6.31	27.90	7.40	0.326
WHR	0.79	0.09	0.84	0.17	0.827

PCOS-polycystic ovary syndrome; HA-hyperandrogenism, BMI-body mass index; HIP-hip circumference; waist-waist circumference; WHR-waist-hip ratio; SD-standard deviation

Supplement 3

Comparison of anthropometric parameters in patients with PCOS and overweight or obesity and in patients with PCOS without overweight or obesity

	PCOS without overweight and obesity *(n=15)*		PCOS with overweight and obesity *(n=14)*		
Parameter	mean	SD	mean	SD	p
Age (year)	24.80	3.32	29.64	9.11	0.169
Hight (m)	1.67	0.05	1.67	0.07	0.810
Weight (kg)	59.87	5.99	89.64	16.22	0.000
HIP (cm)	97.07	6.05	113.14	9.65	0.000
WC (cm)	74.07	8.58	98.79	17.52	0.000
Systolic pressure (mmHg)	113.73	10.10	124.21	16.72	0.050
Diastolic pressure (mmHg)	74.33	9.98	82.29	10.16	0.050
BMI (kg/m^2)	21.53	1.90	32.10	6.04	0.000
WHR	0.76	0.08	0.88	0.16	0.011

PCOS-polycystic ovary syndrome; BMI-body mass index; HIP-hip circumference; WC-waist circumference; WHR-waist-hip ratio; SD-standard deviation
